# Chitosan Nanoparticles for Therapy and Theranostics of Hepatocellular Carcinoma (HCC) and Liver-Targeting

**DOI:** 10.3390/nano10050870

**Published:** 2020-04-30

**Authors:** Maria Cristina Bonferoni, Elisabetta Gavini, Giovanna Rassu, Marcello Maestri, Paolo Giunchedi

**Affiliations:** 1 Department of Drug Sciences, University of Pavia, 27100 Pavia, Italy; cbonferoni@unipv.it; 2 Department of Chemistry and Pharmacy, University of Sassari, 07100 Sassari, Italy; eligav@uniss.it (E.G.); grassu@uniss.it (G.R.); 3 IRCCS Policlinico San Matteo Foundation, General Surgery, 27100 Pavia, Italy; mmaestri@smatteo.pv.it

**Keywords:** nanoparticles, chitosan, HCC, hepatic surgery, theranostics, liver-targeting

## Abstract

Chitosan nanoparticles are well-known delivery systems widely used as polymeric carriers in the field of nanomedicine. Chitosan is a carbohydrate of natural origin: it is a biodegradable, biocompatible, mucoadhesive, polycationic polymer and it is endowed with penetration enhancer properties. Furthermore, it can be easily derivatized. Hepatocellular carcinoma (HCC) represents a remarkable health problem because current therapies, that include surgery, liver transplantation, trans-arterial embolization, chemoembolization and chemotherapy, present significant limitations due to the high risk of recurrence, to a lack of drug selectivity and to other serious side effects. Therefore, there is the need for new therapeutic strategies and for improving the liver-targeting to HCC. Nanomedicine consists in the use of nanoscale carriers as delivery systems to target and deliver drugs and/or diagnostic agents to specific organs or tissues. Chitosan and its derivatives can be successfully used in the preparation of nanoparticles that, for their peculiar surface-properties, can specifically interact with liver tumor, by passive and active targeting. This review concerns the use of chitosan nanoparticles for the therapy and theranostics of HCC and liver-targeting.

## 1. Introduction

Hepatocellular carcinoma (HCC) represents a global health problem because it is one of the most common causes of death due to tumoral diseases [1]. It is possible to identify different risk factors involved in its etiology: in western countries and Japan its diffusion is in relation with hepatitis C virus, chronic alcohol abuse, non-alcoholic fatty liver disease and steatohepatitis by metabolic syndrome, while in Africa and Asia is mainly due to infections by hepatitis B virus and to aflatoxin B1 consumptions [2].

**Figure 1:**
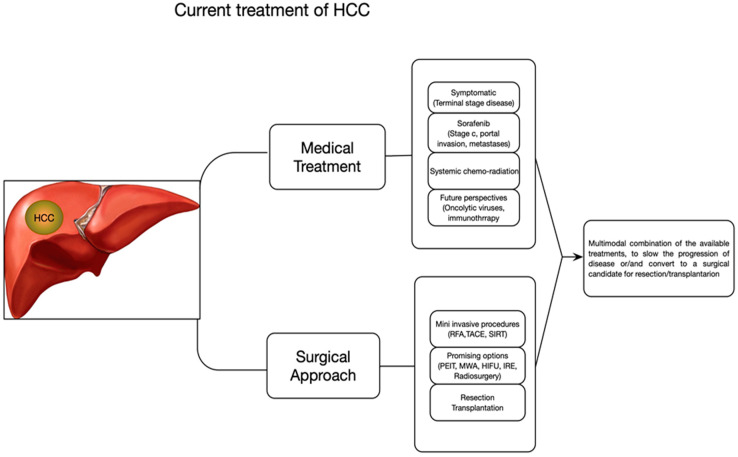
Therapeutic strategies for hepatocellular carcinoma (HCC).

Although in the last years some advances have been made both in early diagnosis and treatments, HCC can have an unfavorable prognosis, and mortality of patients is still high. Different therapeutic strategies are available for the therapy of HCC (Figure 1).

**Figure 2:**
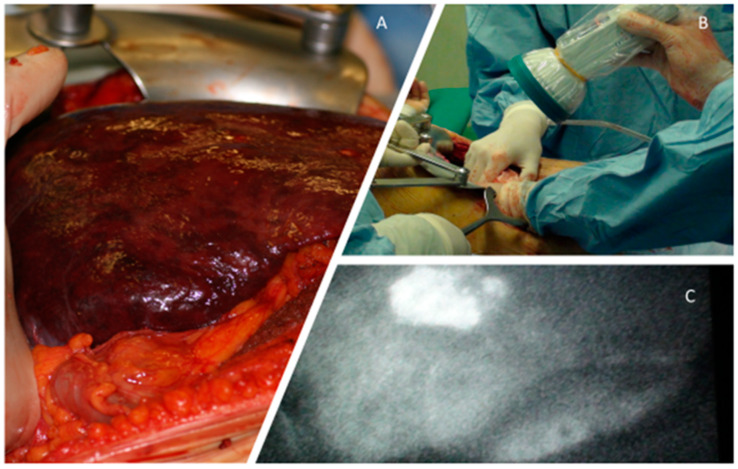
Cirrhotic liver (**A**). An intraoperative near-infrared fluorescence (NIR) examination (**B**) confirms an HCC (**C**).

Among these therapeutic options the HCC surgical approach is, whenever possible, the “first choice”, with hepatic resection aimed to a precise removal of cancerous tissues. In this perspective there are many methods of tumor visualization by intraoperative near-infrared fluorescence (NIR) imaging (Figure 2) [3,4,5,6,7]. Liver transplantation is, in some cases, necessary [8,9,10].

The success of the curative effects obtained by hepatic resection and liver transplantation is however to some degree limited by the risk of postoperative HCC recurrence and metastasis. Furthermore, relatively few patients are candidates for surgical therapy, owing to metastasis at the time of diagnosis and to the lack of transplantable livers [11]. Other possible options are trans-arterial (chemo)embolization and radiotherapy [12,13,14,15,16,17,18]. Chemotherapy efficacy is limited due to drug resistance, lack of selectivity and serious side effects. In addition, many of the anticancer drugs currently available are characterized by short plasmatic half-life and low water-solubility, and consequently their therapeutic efficacy can be limited by low and irregular bioavailability [19].

Therefore, the investigation for new therapeutic strategies involving liver-targeting and early-diagnostic methods is an important challenge.

Nanomedicine is based on the peculiar properties of nanoscale carriers that are designed to target and deliver drugs and/or diagnostic agents to specific organs and tissues [20,21,22,23,24]. Therefore, nanotechnology has important applications in cancer diagnosis and therapy [25,26,27,28]. Different kinds of nanocarriers can be used. Polymeric nanoparticles, which are based on polymers that must be safe, biocompatible and biodegradable, have remarkable importance [24,29,30,31]. These nanoparticles are able to control drug release and to enhance drug stability; furthermore, they improve drug accumulation in the target tumor because they can specifically enter neoplastic sites through passive and/or active pathways therefore reducing drug toxicity [32].

Passive targeting is related to the size of nanoparticles that during circulation can easily penetrate the tumor vessels. Nanoparticles with the size of 10–500 nm are in fact well-known to pass through fenestrated vessels that characterize the tumor lesions; the consequent enhanced permeability and retention (EPR) effect leads to the specific accumulation of the nanoparticles into the tumors. [33]. Active targeting utilizes ligand-receptor interactions [32]. In the case of active targeting, functional ligands, that are capable to interact with receptors overexpressed on the surface of the desired target cells, are linked onto the surface of nanoparticles via chemical coupling or physical coating.

**Figure 3:**
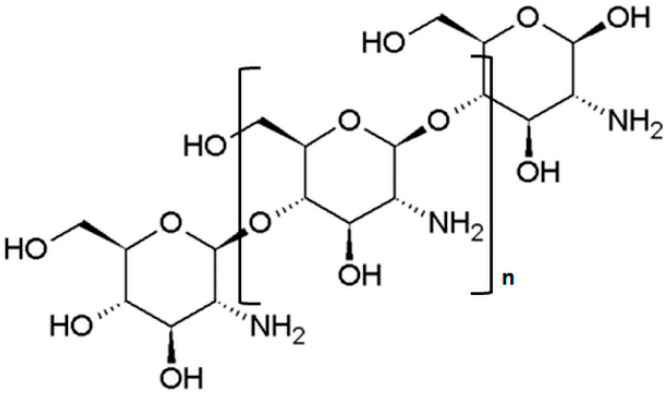
Chemical structure of chitosan.

Chitosan is a linear amino-polysaccharide (poly-1,4-D-glucosamine) obtained by a partial deacetylation of chitin, which is a component of the exoskeleton of insects and crustaceans and it is one of the most abundant polysaccharides present in nature (Figure 3).

It is a polycation due to its amino groups which are ionized in weakly acidic environments, allowing the polymer to interact with negatively charged surfaces, e.g., cell membranes [34,35,36]. In the pharmaceutical field, chitosan is used because of its high biocompatibility, biodegradability, and low toxicity. Furthermore, it is characterized by mucoadhesive and penetration enhancer properties that make it a material with significant potential [37,38,39,40]. It is therefore widely used for the preparation of drug formulations administered through different routes: oral, parenteral, transmucosal [41,42,43,44,45,46]. Owing to the chemical characteristics of the polymer, chitosan nanoparticles have a surface with a positive charge that provides affinity for negatively charged biological membranes [47]. It has been demonstrated that they accumulate selectively in tumor and not in the normal tissues because of EPR effect [48] (passive targeting).

Recently, this polymer has been used for the preparation of gene delivery carriers because chitosan is able to interact with DNA fragments and at acidic and neutral pHs it condenses DNA to nanoparticles, due to the presence of the amino groups [49]. Chitosan nanoparticles have also been used as a drug delivery system for therapeutic treatments of pancreatic cancer [50]. Furthermore, amino groups make the chitosan derivatization easy [51]. As we can see in many examples of literature reported here below, this characteristic represents a precious tool to link to chitosan structure specific ligands for receptors of especially tumoral cells, obtaining “smart” drug formulations, able to target a specific organ/tissue (active targeting).

The present review is dedicated to nanoparticles made of chitosan and chitosan derivatives, designed for the therapy and theranostics of HCC and liver-targeting. Table 1 reports the most exemplary papers in the last years dealing with unloaded or loaded chitosan nanoparticles.

The main part of the papers here reviewed regards chitosan of commercial origin and sometimes no indication about Mw and deacetylation degree is given. Many of the papers show in vitro, technological characterization of the nanoparticles, all the papers describe studies on cell cultures with hepatic tumor cell lines and in vivo studies on animals, while no clinical study is reported.

## 2. Unloaded Chitosan Nanoparticles for Cancer Therapy: The Anti-Cancer Properties of Chitosan Itself on Hepatocellular Carcinoma (HCC) Cells

As shown by HCC literature, chitosan results “something more” than an excipient or just a basis for nanocarrier formulations: a few researchers, in fact, claimed that unloaded chitosan nanoparticles, by itself, even unloaded, are characterized by anti-tumoral properties with respect to HCC cells. Some of these authors, reported here below, go so far as to say that chitosan should be considered as a molecule with anticancer therapeutic potential. Although to confirm this statement further investigations are required, the biological properties of the polymer certainly make it interesting in the design of a drug loaded anticancer formulation.

One of the first authors that demonstrated the antitumor activity of chitosan nanoparticles was Qi et al. [52,53]. The anti-cancer effect has been studied on human hepatoma cells BEL7402 [52]: the cells were grown in the presence and in the absence of different concentrations of chitosan nanoparticles (sizes in the range 40–100 nm). Cell viability and surface charge were studied. In vitro, chitosan nanoparticles showed their anti-tumor properties acting at different levels to determine the death of HCC cells: they induced cell necrosis, reduced cell viability, neutralized the negative surface charge thanks to the positive charge of the polymer, modified the fatty acid composition of the membranes and determined the fragmentation of DNA. Furthermore, in vivo experiments were carried out on animals (male BABL/c nude mice), which were subcutaneously implanted with BEL7402 cells. Chitosan, saline and nanoparticles with different mean particle size were orally administrated (1 mg/kg body weight). Tumor and body weights were measured, the morphologic changes of tumor and liver tissues were studied using SEM. In nude mice implanted with BEL7402 cells and treated with chitosan nanoparticles with different mean particle sizes (40, 70 and 100 nm), the tumor growth inhibitory rates increased with the decrease of mean particle size of the nanoparticles. No liver abnormalities were found under electron microscope. The antitumor properties of chitosan nanoparticles were studied by Qi et al. also on mouse hepatoma H22 bearing mice [53]. These studies showed that chitosan nanoparticles, administered by intravenous injections, are characterized by antitumor activity, which results higher for smaller particle sizes. The in vivo efficacy is related by the authors, also in this case, to the positive charge and to the particle size of the nanoparticles.

Further studies on chitosan nanoparticles were carried out by Xu et al., also in this case using a model of nude mice xenografted with BEL7402 cells [54]. The nanoparticles were prepared by the ionotropic gelation of cationic chitosan with polyanion sodium tripolyphosphate. Chitosan nanoparticles were given by oral administration once daily to groups of six animals. The results demonstrated that the treatment significantly inhibited the growth of the tumor and induced its necrosis. Furthermore, determination of micro vessel density suggested that these results are correlated with the inhibition of tumor angiogenesis. This is in accordance with the results obtained by Harish-Prashanth and Tharanathan [55] that demonstrated that chitosan inhibits angiogenesis in vitro. For these promising results the authors come to affirm that chitosan nanoparticles should be considered as a novel class of anti-cancer drugs.

More recently, anti-carcinogenic and hepato-protective properties of nanoparticles based on chitosan extracted from the gladius of squid *Sepioteuthis lessoniana*, were found on diethylnitrosamine-induced HCC, in male Wistar rats [56]. The study showed that the supplementation of the rats with the chitosan nanoparticles had a protective effect on liver cells evidenced by the reduction of the levels of marker enzymes and bilirubin and thus increasing the albumin levels. The treatment with the chitosan nanoparticles led also to an enhancement of the levels of antioxidant enzymes, and lipid peroxidation products were diminished. The authors claimed that the protective effect of chitosan nanoparticles on hepatic cells could be related to the counteraction of free radicals and to the inhibition of the lipid peroxidation. They claimed that chitosan could be a promising drug for treating HCC in future and clinical trials are further needed.

Elkeiy et al. studied the effect of chitosan nanoparticles against HCC both in vitro (HepG2 cells) and in vivo (diethylnitrosamine-induced HCC in rats) [57], using chitosan extracted from the phototropic nauplii (larvae developed from hatched *Artemia salina* eggs). The results of this study showed that chitosan nanoparticles inhibited HCC progression in vitro (on HepG2 cells) and in vivo, with the induction of necrosis, mainly by damaging and disrupting the membranes of the cells. The in vivo experiments showed that the pre-treatment of the animals with the nanoparticles had better therapeutic effects with respect to those obtained with the post-treatment.

A further study has been carried out with chitosan nanoparticles against diethylnitrosamine-induced HCC in rats [58]. Chitosan nanoparticles showed a significant modulatory effect on proinflammatory cytokines, oxidative stress and apoptosis and for these reasons they can be considered as useful tools in an innovative cancer therapy.

## 3. Drug Loaded Chitosan Nanoparticles

In all the papers above reported, chitosan itself is used in unloaded nanoparticles, as the only “active component”. However, in many other papers it is proposed as the basis of nanocarriers loaded with different actives for therapeutic or theranostic purposes. In all these cases the liver-targeting ability of chitosan nanoparticles has been pointed out, and it is mainly related to the size and the surface properties of the polymer.

Yang et al. prepared chitosan nanoparticles (average particle size of about 175 nm, chitosan with deacetylation degree of 93%) loaded with 125I-labeled 5-iodo-2′-deoxyuridine [59]. It was found that the nanoparticle accumulation was significantly higher in HCC cells HepG2 than in normal liver cells HL-7702. In vivo animal experiments were carried out on Male New Zealand white rabbit VX2 liver tumor. According to the authors, the way by which the drug loaded chitosan nanoparticles come into the tumor is mainly represented by passive targeting. These experiments showed that the internal irradiation obtained by drug loaded nanoparticles, induced significant cell apoptosis and enhanced DNA-damage in rabbit hepatocellular tumors, with respect ^125^I-labeled 5-iodo-2′-deoxyuridine infusion at the same dose.

Hydroxycamptothecin (HCPT) loaded chitosan nanospheres (average size of about 330 nm) were prepared using the membrane emulsification technique [60]. HCPT is an alkaloid isolated from the *Camptotheca acuminate* in China. It shows anticancer activity although the clinical application is limited owing to its water-insolubility. SEM images showed that drug loaded nanospheres have spherical morphology with a size of 200–300 nm. The nanospheres exhibited in vitro an initial burst effect followed by a sustained HPCT release of more than 15 days. An anti-tumor ability was observed towards human hepatoma (HepG2) cells. In vivo study was carried out by injecting subcutaneously intra-tumor, the drug loaded HCPT nanoparticles into mice bearing HepG2 tumor and put into evidence that the nanoparticles reduced tumor weight and growth rate.

CD147 antibody was coupled with α-hederin loaded chitosan nanoparticles by Zhu et al. [61]. The chitosan had a Mw of 8 kDa–10 kDa and deacetylation degree of 90.9%. α-hederin is a saponin extracted from *Pulsatilla chinensis* (Bge.) Regel, exhibiting cytotoxicity and inducing apoptosis of cancer cells. CD147 is an antibody that can induce tumor necrosis. The nanoparticles had an average particle size of about 150 nm. In vitro cytotoxicity assays were performed on HepG2 cells that after 2 h of incubation exhibited a significantly high uptake of nanoparticles, through clathrin-mediated endocytosis.

Doxorubicin is one of the most important anticancer drugs with broad-spectrum antineoplastic activity, but despite its antitumor properties adverse effects such as cardiotoxicity have limited its utility. For this reason, Ye et al. prepared chitosan doxorubicin nanoparticles as drug delivery system in liver cancer [62]. Chitosan doxorubicin nanoparticles (average size of 30–40 nm) inhibit the growth of HepG2 cells by promoting apoptosis and arresting cell cycle at G2/M phase.

Epirubicin is one of the main line treatments for HCC, but in case of long-term administration it has severe side-effects including cardiomyopathy and congestive heart failure. For this reason, Nasr et al. prepared nanoparticles made by a combination of chitosan and PLGA, absorbed with asialofetuin, a glycoprotein displaying affinity for asialoglycoprotein receptor expressed on the surface of parenchymal hepatocytes, to achieve the specific targeting to hepatocytes [63]. Drug loaded chitosan-PLGA nanoparticles were prepared by an emulsion diffusion technique. The asialofetuin absorption process onto the chitosan-PLGA nanoparticle surface was made at 4 °C for 18 h. Drug loaded nanoparticles injected in HCC mouse model revealed higher p53-mediated apoptosis and reduced angiogenesis in the tumor. The authors claimed that chitosan probably imparted stealth properties to the particles leading to a prolonged circulation, a reduced opsonisation and phagocytosis. This statement is according to similar considerations made by Sarmento et al. [66]. The combination of drug loaded chitosan-PLGA nanoparticles with tocotrienols further enhanced apoptosis and reduced VEGF levels [63].

Dhanapal et al. prepared chitosan (Mw 90 kDa)/PLA nanoparticles loaded with piceatannol, a polyphenol present in red wine with anti-inflammatory, antioxidant and anti-leukemic properties [64]. Drug loaded chitosan-PLA nanoparticles were incubated at different concentrations (1, 5, 10, 20 and 40 mg/mL) with HepG2 cells. The results of cell viability assays showed a cytotoxic effect towards cancer cells in a dose-dependent manner and apoptosis in mitochondria-dependent pathways.

Chitosan nanoparticles have been used also for theranostics purposes. In an interesting study chitosan was used to coat superparamagnetic iron oxide nanoparticles (SPIONs) to prepare contrast agents in magnetic resonance imaging (MRI) [65]: the interest of these particles is growing owing to their importance in the theranostics. In fact, they are able to enhance magnetic resonance imaging (MRI) signals, thus permitting more accurate diagnosis and visualization and at the same time they can be used as drug carriers for a specific targeting. To study the biodistribution of SPION-coated chitosan nanoparticles, 8-week-old female BALB/c mice, specific pathogen free were used. The results indicate that nanoparticles accumulate in kidneys and liver as they are the organs mainly involved in the clearance of SPIONs. Thus, these experimental results show that chitosan coated SPION nanoparticles are promising contrast agents for diagnosis of liver diseases.

## 4. Chitosan Derivatives for Drug Loaded Nanocarrier Preparation

In literature it is described the use of chitosan derivatives for the preparation of nanoparticles that can be potentially useful in HCC therapies and liver-targeting. The processes of chemical modification of chitosan usually involve the free amino groups of its deacetylated units. These modifications can be made to modify/improve some specific characteristics of the polymer, such as the hydrophilic character, but they are principally aimed to impart a specific affinity towards bioactive molecules and towards receptors over-expressed in tumor cells, to achieve a selective tumor targeting. Here below are reported some of the most interesting examples.

### 4.1. Galactosylated Chitosans

**Figure 4:**
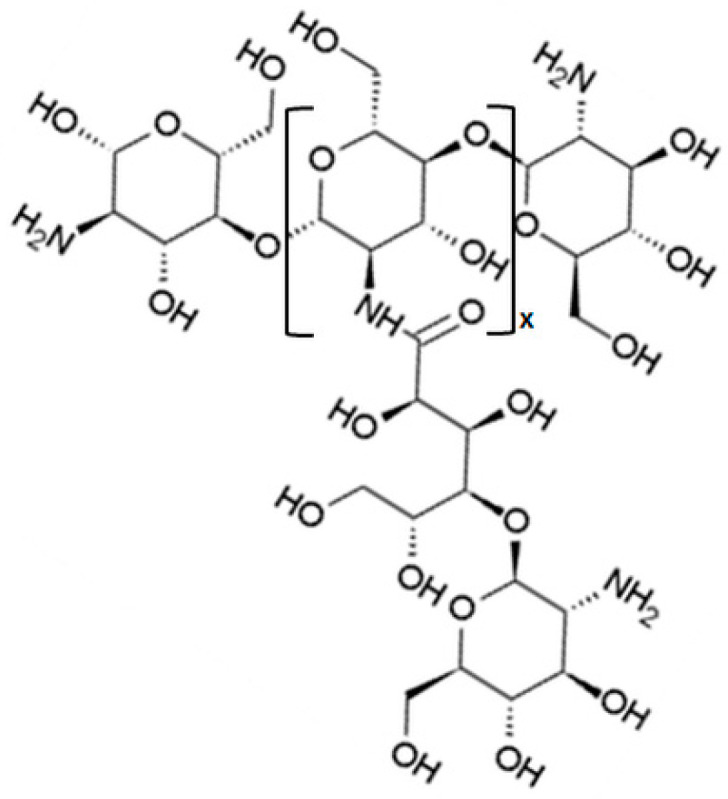
Galactosylated chitosan.

Galactosylated chitosan (Figure 4) can be used for the preparation of nanoparticles capable of active targeting (Table 2). Galactose is among the few selective ligands used for targeting HCCs due to its high binding affinity to asialoglycoprotein receptors (ASGPR) overexpressed in hepatocytes and HCCs [67,68]. ASGPRs is present in abundance on hepatocytes, it is minimally expressed on extrahepatic tissues and it could provide a way to reach hepatocyte-mediated delivery [68].

One of the first examples of the use of galactosylated chitosan is by Kim et al. [69] that designed a nanocarrier based on galactosylated chitosan, for gene delivery into hepatic cells. The liver is characterized by a rich blood supply that can allow the delivery of genes to it and then their distribution to the systemic circulation. The success of gene therapy is related to the design of delivery systems that can release the therapeutic genes into the target cells. Starting from these observations, galactosylated chitosan was complexed with plasmid DNA in various charge ratios. Cytotoxicity and transfection efficiency of DNA complexed galactosylated chitosan were studied in cultured HepG2 cell line. Galactosylated chitosan exhibited much enhanced gene transfer efficiency on HepG2 cells than chitosan itself. The high selectivity of the galactosylated chitosan nanoparticles for the liver was associated with low cytotoxicity.

Starting from this interesting work, it was developed the idea of using galactosylated chitosan for the therapy of HCC. Cheng et al. developed galactosylated chitosan nanoparticles containing 5-fluorouracil, a cytotoxic drug used for the treatment of solid tumors such as liver cancer [70,71,72]. 5-fluorouracil is rapidly metabolized, and its long-term therapy is associated with some important side effects. Drug delivery systems able to give prolonged drug release in the site of tumor can be a good approach to improve therapy. Galactosylated chitosan nanoparticles loaded with 5-fluorouracil have been prepared, to exploit the specific affinity of the chitosan derivative with the asialoglycoprotein receptors. The nanoparticles were tested in vitro and on liver cancer in vivo. The results obtained indicated that galactosylated chitosan is a good polymer for the preparation of 5-fluorouracil loaded nanoparticles, that are characterized by a sustained drug release capacity. Drug loaded nanoparticles inhibited tumor growth in an in vivo model of liver cancer (mouse) more markedly than 5-fluorouracil alone [70,71,72].

Nanoparticles composed of galactosylated chitosan oligosaccharide and adenosine triphosphate (ATP) were prepared by Zhu et al. and it was demonstrated that these nanoparticles are up taken by HepG2 cells, due to expression of the asialoglycoprotein receptor on their surfaces [73].

Galactosylated chitosan (galactosylation degree of 10.3%)/polycaprolactone nanoparticles for hepatocyte-targeted delivery of curcumin were prepared by Zhou et al. [74]. Curcumin is a polyphenol obtained by extraction from Curcuma longa plant and it is used for its antioxidant, anti-inflammatory and anticancer properties; the mechanisms of action of the anticancer activity is apoptosis and inhibition of proliferation of tumors by suppressing signaling pathways [75]. However, the use of curcumin is limited by its low water solubility and consequent poor bioavailability. Polycaprolactone has been grafted onto chitosan backbone to obtain an amphiphilic copolymer that can be used for the preparation of nanoparticles able to deliver the water-insoluble curcumin. The galactosylation of the copolymeric nanoparticles was made to achieve hepatocyte-targeted specificity. Experiments showed that nanoparticles had the ability to induce in HepG2 cells an apoptosis 6-fold higher than in cells treated with free curcumin. These results suggest that the galactosylated chitosan-polycaprolactone nanoparticles are promising polymeric carriers for liver-targeted delivery of curcumin.

Galactosylated-carboxymethyl chitosan-magnetic iron oxide nanoparticles were prepared by Xue et al. [76] and conjugated to the surface with galactose: in vitro experiments showed that these nanoparticles are highly selective for HCC cells, while in vivo experiments put in evidence their accumulation in HCC tissue, especially when an external magnetic field is applied. Nude mice were transplanted with HCC and then received an intravenous injection of the nanoparticles loaded with RASSF1A, a tumor suppressor gene. Transfection of HCC cells with the RASSF1A gene nanoparticles inhibited the growth of tumors and increased the sensitivity of HCC cells to chemotherapy.

Galactosylated-chitosan nanoparticles were prepared and loaded with triptolide, an active component derived from a Chinese herb (*Tripterygium wilfordii* Hook F) that may possess anti-inflammatory and anti-cancer effects [77] However, triptolide has low water solubility and high toxicity and this limits its clinical use. In vivo experiments carried out on mice showed that free triptolide prevents cancer progression at the cost of high toxicity, while the loading into of the drug into nanoparticles determines an improvement of drug efficacy, and at the same time lowers its systemic toxicity. The main reason of this result is due to the combination of sustained drug release and liver-targeted delivery determined by the nanoparticles.

Recently, galactosylated-chitosan nanoparticles have been proposed as carriers for HCC liver targeting of gemcitabine, an antimetabolite pyrimidine nucleoside derivative [78]. The in vitro release tests showed a prolonged drug release from the nanoparticles. In vivo experiments carried out on an HCC rat model showed that the clearance of gemcitabine in the plasma is rapid from nanoparticles as compared to drug alone. Organ distribution data demonstrated a good liver targeting.

### 4.2. Glycyrrhizin/Glycyrrhetinic Acid Conjugated Chitosans

**Figure 5:**
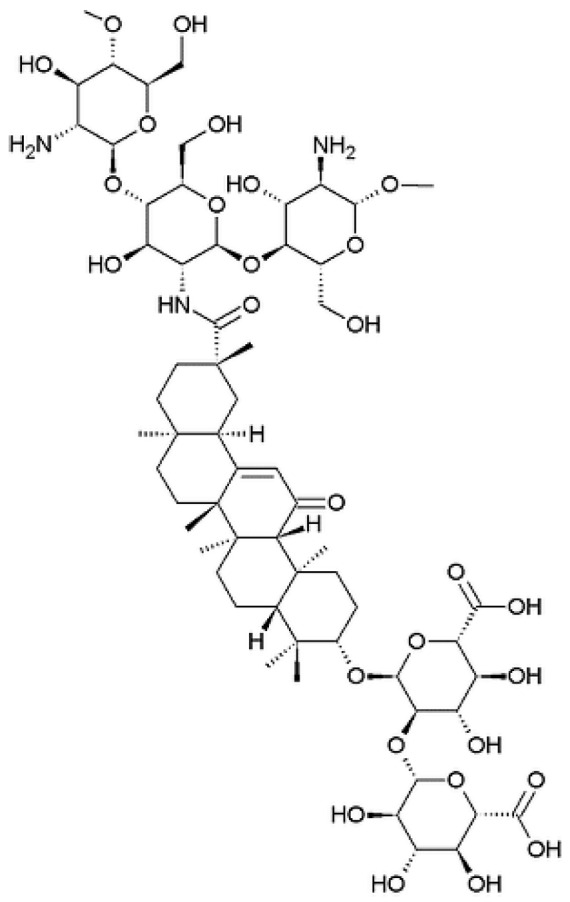
Glycyrrhizin conjugated chitosan.

The use of glycyrrhizin conjugated chitosan (Figure 5) represents another example of receptor-mediated liver targeting strategy (Table 3). Glycyrrhizin and glycyrrhetinic acid are the principal compounds extracted from the root of Glycyrrhiza glabra (licorice). These substances are used for their anti-hepatitis and anti-hepatotoxic effects [79,80]. It has been demonstrated that there are specific binding sites of glycyrrhizin and glycyrrhetinic acid on the cellular membrane of rat hepatocytes [81]. The discovery of these new ligands for the targeting of the liver is, of course, highly relevant because it gives new tools for HCC therapy.

Among the first researchers that used glycyrrhizin derivatives of chitosan for the preparation of nanoparticles characterized by active hepatic targeting were Lin et al. that prepared chitosan nanoparticles surface-modified with glycyrrhizin [81]. Their studies showed that the nanoparticles preferentially accumulated in hepatocytes. Starting from this work, several researches have been developed to achieve liver targeting that can be useful in the therapies of liver pathologies in general and in particular of HCC.

Mishra et al. [82] obtained low-molecular weight chitosan from high-molecular weight chitosan through depolymerization, and then prepared, by ionotropic gelation method, nanoparticles that were conjugated with glycyrrhizin and loaded with lamivudine and fluorescein isothiocyanate, to obtain at the same time hepatic-targeting and controlled drug release. Lamivudine is a drug of the group of non-nucleoside reverse transcriptase inhibitors, with anti-retroviral properties. In vivo organ biodistribution studies carried out on albino rats showed that the loading of lamivudine in glycyrrhizin conjugated chitosan nanoparticles enhances hepatic residence time as well as concentration of drug in the liver.

Glycyrrhetinic acid–chitosan nanoparticles for liver targeting of atorvastatin have been prepared by Rohilla et al. [83]. Atorvastatin, a statin, is used for the lowering of cholesterol in coronary heart disease: liver targeting of atorvastatin minimizes the drug level in systemic circulation, and therefore its toxicity. Pharmacokinetic parameters were studied after oral administration of atorvastatin loaded both glycyrrhetinic acid–chitosan nanoparticles and chitosan nanoparticles, in a Wistar rats model. The results of in vivo studies showed that by specifically delivering the drug to the liver, high reduction in hepatotoxicity was achieved compared to the chitosan nanoparticles and this can be useful in the therapy of HCC.

Glycyrrhetinic acid conjugated chitosan/PEG (poly(ethylene glycol) nanoparticles for the targeting of liver were prepared by Tian et al. [84]. In their study glycyrrhetinic acid was chosen as the targeting ligand. The nanoparticles were obtained by an ionic gelation process. The chitosan raw material had a degree of deacetylation >95% and Mw of 50,000. The cellular uptake was studied using HCC cells (QGY-7703 cells). The anti-neoplastic properties of doxorubicin.HCl-loaded nanoparticles was studied in vivo using Balb/c nude mice which were injected subcutaneously in the right limb armpits with 0.1 mL of a cell suspension containing 1 × 10^6^ mouse H22 hepatoma cells. The results showed that the nanoparticles are successfully targeted to the liver, the accumulation in the liver was 51.3% at 3 h after injection (nearly 2.6 times that obtained with not conjugated chitosan nanoparticles). Furthermore, the presence of glycyrrhetinic acid conjugated chitosan significantly increased the affinity to human hepatic carcinoma cells (QGY-7703 cells) with approximately a 19-fold improvement in cellular uptake. The drug loaded nanoparticles were remarkably cytotoxic to QGY-7703 cell lines and they could effectively inhibit tumor growth in H22 cell-bearing mice.

Lin et al. prepared glycyrrhizin conjugated N-caproyl chitosan nanoparticles and showed that they enhanced in vivo rat hepatocyte uptake by 2.1 folds compared to not derivatized N-caproyl chitosan nanoparticles [85]. The final product was constituted by particles of spherical shape, whose size was of 100–205 nm. Drug encapsulation efficiency was of more than 83%. Glycyrrhizin modified N-caproyl chitosan nanoparticles determined the internalization of liver cancer SMMC-7721 cells by approximately 10-fold as compared to unconjugated nanoparticles. Biodistribution experiments carried out on mice with induced hepatoma H22 ascites showed that drug loaded glycyrrhizin conjugated N-caproyl chitosan nanoparticles have significantly higher targeting to tumor than drug loaded chitosan nanoparticles. Some years later, glycyrrhizin conjugated O-carboxymethyl (CM) chitosan nanoparticles have been prepared and loaded with paclitaxel, chosen as a model antitumor drug [86].

Finally, dual-ligand glycyrrhetinic acid and galactose-modified chitosan nanoparticles were recently prepared by Li et al. [87]. The authors reported that the carrier constituted by this modified chitosan was able to specifically internalize hepatoma cells in vitro and to accumulate into HCC in vivo.

### 4.3. Other Chitosan Derivatives

Other chitosan derivatives are used for the preparation of nanoparticulate systems for HCC therapies and liver-targeting (Table 4).

#### 4.3.1. Folate-Conjugated Chitosans

**Figure 6:**
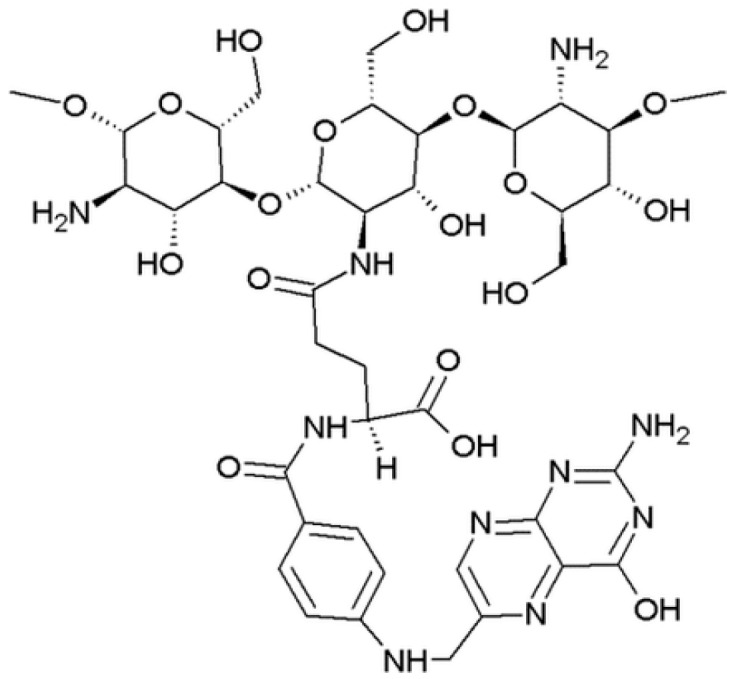
Folate-conjugated chitosan.

Liver gene therapy has been carried out as an alternative therapy for many liver diseases, including liver cancer. However, this therapy needs safe and effective gene carriers to overcome the problems of the use of “naked” genes, such as poor cellular uptake, extensive enzyme degradation, and rapid clearance by renal filtration. Folate-conjugated chitosan (Figure 6) has been proposed as polymer for the preparation of nanoparticles for gene therapy of HCC. In fact, it is demonstrated that tumor cells are characterized by a higher density of folate (FA) receptors compared to normal cells. Starting from this observation, Hu et al. [88] prepared folate-conjugate chitosan (deacetylation degree ≥90%) nanoparticles and loaded them with the expression plasmid of the mouse interferon-induced protein-10 (IP-10) gene, a potent chemoattractant for cytotoxic T cells.

Folate-conjugated chitosan nanoparticles were prepared and loaded with mouse IP-10 gene, for HCC immunotherapy by Lai et al. [89]. In vivo experiments were carried out using mice bearing H22 tumor cells treated with the nanoparticles containing IP-10 gene. Treatment of H22 tumor-bearing mice with the nanoparticles led to inhibition of angiogenesis, promotion of IP-10 expression and induction of apoptosis in the tumors.

Folate-PEG-chitosan-graft-low molecular weight polyethyleneimine was used for the preparation of nanoparticles by Kim et al. [90] and employed as a potential gene carrier for the targeting to cancer cells characterized by over-expressed folate receptors. In vivo experiments were carried out on H-ras12V mice with liver cancer, showing that these polymeric nanoparticles have high tumor cell specificity.

#### 4.3.2. Biotinylated Chitosans

**Figure 7:**
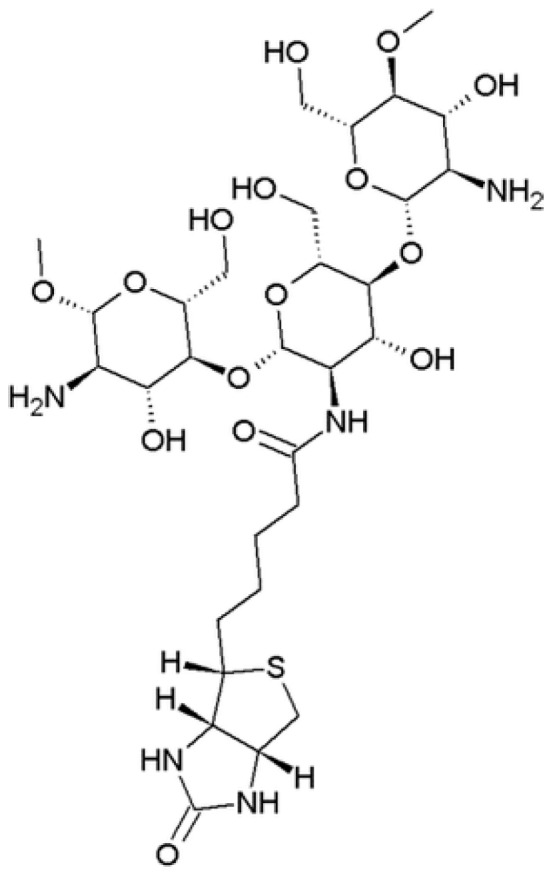
Biotinylated chitosan.

Biotin (vitamin H) is a small molecule that presents a specific receptor that can be found on the surface of almost all cells of the body. Biotin receptors are, however, over-expressed in certain cancer cells in comparison of normal cells; for example, biotin adsorption in hepatoma cells has been demonstrated to be about 40 times higher compared with that the adsorption in normal hepatocytes [91,92]. This over-expression is due to the large quantity of nutrients that the rapid/excessive tumor growth needs. Starting from these considerations, biotinylated chitosan (Figure 7) nanoparticles of mean particle size of about 83 nm have been prepared by Cheng et al. for the targeting of liver tumor [93]. The degree of substitution of biotin was 35%, the nanoparticles were loaded with plasmid DNA in a molar ratio 1:1 with the polymer. Confocal laser scanning and in vivo imaging assays carried out on biotinylated chitosan nanoparticles demonstrated the liver cancer targeting using human HCC cells (SMMC-7721) and a liver cancer mouse model, compared with the “naked” plasmid DNA.

With the aim to target HCC, chitosan nanoparticles decorated with biotin alone or with biotin and avidin, and loaded with trans-resveratrol, chosen as a model anti-cancer drug, were prepared [94]. Avidin is a highly glycosylated protein that includes various alkaline amino acids, such as lysine and arginine: when avidin is injected intraperitoneally, it accumulates in the abdominal tumor. The antitumor activity was examined with inhibitory studies on HepG2 cells showing that with respect to trans-resveratrol solution, both biotin and biotin/avidin nanoparticles remarkably enhanced the anticancer activity.

#### 4.3.3. N,N,N-Trimethyl/Alkyl Chitosans

Chitosan can be derivatized by N-methylation to form N,N,N-trimethyl chitosan (TM-chitosan) chloride. This polymer is very interesting for its surface properties because thanks to its quaternary ammonium groups and the strong positive charge, it can form stable complexes with anionic compounds such as drugs, proteins and DNA fragments that can be utilized for HCC liver-targeting. Monoclonal antibodies specific to tumor antigens or tumor-associated antigens represent a new opportunity for the treatment of tumors: for example, mouse monoclonal antibodies against HCC have been used for tumor imaging and targeting [95]. However possible problems are clinical toxicity and high immunogenicity that can induce the production of human anti-mouse antibodies. Vongchan et al. [96] prepared a monoclonal antibody, active against human liver heparan sulfate proteoglycans which are implicated in tumor biology (they mediate adhesion and migration of tumor cells and responses to mitogenic and angiogenic growth factors) and therefore inhibiting HCC. To overcome the problems of toxicity and immunogenicity of this monoclonal antibody, it was formulated as nanocomplexes with TM-chitosan. The polymer (Mw of 276 kDa), in fact, formed nanocomplexes by electrostatic interaction with the carboxylate group of the monoclonal antibody, giving nanoparticles of spherical shape and with a size of about 59 nm. Cellular uptake of fluorescent-labeled nanoparticles was studied using mouse monocyte models of cancer and normal cells (mouse monocyte cell lines, J774). These experiments showed that the TM-chitosan nanoparticles enter both normal and cancer HepG2 cells, but they are retained in HepG2 cells for a longer period of time and exhibit greater toxicity.

Guan et al. prepared TM-chitosan nanoparticles containing lactosyl-norcantharidin, for liver cancer therapy [97,98]. The polymer had a Mw of 8–30 kDa. According to the authors, their choice of the quaternized cationic TM-chitosan for the preparation of nanoparticles provided strong electrostatic interaction with negatively charged tumors improving the absorption-enhancing properties of the polymer [99]. Norcantharidin is an anti-cancer drug, lactosyl-norcantharidin is the corresponding prodrug, bearing a galactose group to achieve active targeting by interaction with the asialoglycoprotein receptors situated on the hepatocyte membrane. Drug loaded TM-chitosan nanoparticles with an average particle size of about 120 nm were obtained. The nanoparticles induced HepG2 cell death by triggering apoptosis; tumor inhibitory activity in mice bearing H22 hepatoblastoma tumors was demonstrated.

A novel chitosan derivative has been prepared by Zhong et al. combining 2-chloroethylamine hydrochloride and N,N-dimethyl-2-chloroethylamine hydrochloride (MixNCH) [100]. As in the case of TM-chitosan, the introduction of N,N-dimethyl amino residues into the chitosan structure has been made to improve the formation of complexes, in this case with siRNA, and consequently to enhance the efficiency of gene silencing. Nanoparticles were prepared with this modified chitosan, for the intracellular delivery of midkine-siRNA (MK-siRNA) in HCC cells. Midkine is a heparin-binding growth cytokine and it is over-expressed in malignant tumors. Zhong et al. demonstrated that MK-siRNA was able to suppress the growth of HepG2 cells in vitro, but the siRNA was characterized by low intracellular uptake and rapid degradation. The MTS assay demonstrated that the nanoparticles had a gene knockdown effect on the HepG2 proliferation.

N-succinyl chitosan has been utilized by Zhu et al., that prepared nanoparticles loaded with doxorubicin, and then coupled with cholesterol-conjugated siRNA incorporated in a low-density lipoprotein [101]. The in vitro antitumor activity, intracellular uptake, and silencing effects of siRNA were studied. The results showed that N-succinyl chitosan nanoparticles can be considered as carriers for tumor drug targeting to the liver for their ability to deliver both siRNAs and the antitumoral drug.

An amphiphilic chitosan was prepared by a derivatization process, obtaining myristoyl- carboxymethyl-chitosan; this polymer was then combined with lactose that has been demonstrated to have a ligand targeting function [102]. Adriamycin-loaded lactose myristoyl- carboxymethyl-chitosan nanoparticles were then prepared [103] to obtain drug targeting for the therapy of HCC. In vitro experiments, on HU7 human hepatocellular carcinoma cells, and in vivo studies, using a subcutaneous H22 xenograft tumor model in mouse were performed. They demonstrated that the polymeric nanoparticles were readily taken up by HU7 cells and in vivo had significant antitumor efficacy with a 62.7% inhibition rate.

Glycol chitosan-5β-cholanic acid nanoparticles were prepared by Na et al. [104] chemically modifying the hydrophilic glycol chitosan with the hydrophobic 5β-cholanic acid. Subsequently, the glycol chitosan-5β-cholanic acid conjugates were labeled with the NIRF dye, Cy5.5. Tumor cell uptake of nanoparticles was studied in vitro using HT29 cells at various pHs, ranging from 5.5 to 8.0. In vivo tests were made with animal models, which were prepared using athymic nude mice and by injection of HT29 cells into the left lobe of the liver. Cy5.5- labeled nanoparticles were injected intravenously and in vivo biodistribution and tumor targeting efficiency of nanoparticles were studied using (noninvasive) fluorescence imaging. The results obtained by in vitro experiments showed that the acidic pH significantly improved the cellular uptake of chitosan nanoparticles, and this was due to the protonation of amine groups the polymer. The high ability of the chitosan nanoparticles to target the tumor in liver tumor bearing mice was confirmed by different in vivo and ex vivo NIRF images and TEMs. These results showed high potentialities of this amphiphilic chitosan derivative for liver-targeting. The authors put in evidence that in their experiments the chitosan nanoparticles were administered intravenously, but in other works glycol chitosan nanoparticles were successfully employed for the delivery of peptides to the blood and brain using the oral route [105,106].

## 5. Conclusions

The present review put in evidence that nanoparticles made of chitosan and chitosan derivatives, proposed for the therapy and theranostics of hepatocellular carcinoma and liver-targeting, have good potentialities to overcome the limitations of the current HCC therapies. This is mainly attributable to chitosan biological properties and to nanoparticle derivatization with ligands leading to a specific liver targeting. These nanosystems have been designed both for therapeutics and for theranostics purposes. In many cases the studies on cells cultures and, in vivo, on animals gave promising results. Clinic studies are however desirable to confirm these results. Future perspectives will involve the research of new chitosan derivatives with improved targeting properties to be used both for diagnosis and in the early phases of the disease

## Figures and Tables

**Table 1:** Chitosan nanoparticles designed for the treatment of HCC and liver-targeting: the therapeutic/theranostic effect and cell cultures used in the study are reported for each reference.

Carrier	Payload	Effect	Cell Culture	Reference
Chitosan	-	anti-cancer	BEL7402; H22	[52,53]
Chitosan	-	anti-cancer	BEL7402	[54,55]
Chitosan	-	anti-cancer inhibition of lipid peroxidation	-	[56]
Chitosan	-	anti-cancer	HepG2	[57]
Chitosan	-	anti-cancer	-	[58]
Chitosan	^125^I-labeled 5-Iodo-2′deoxyuridine	anti-cancer	HepG2	[59]
Chitosan	hydroxycamptothecin	anti-cancer	HepG2	[60]
Chitosan	CD147 antibody	anti-cancer tumor necrosis	HepG2	[61]
Chitosan	doxorubicin	anti-cancer	HepG2	[62]
Chitosan-PLGA	epirubicin	anti-cancer anti-angiogenic	-	[63]
Chitosan-PLA	piceatannol	anti-cancer anti-oxidant	HepG2	[64]
Chitosan iron oxide	SPIONs	magnetic resonance imaging(MRI)	-	[65]

**Table 2:** Galactosylated-chitosan nanoparticles designed for the treatment of HCC and liver-targeting: the therapeutic/theranostic effect and cell cultures used in the study are reported for each reference.

Carrier	Payload	Effect	Cell Culture	Reference
Galactosylated chitosan	plasmid DNA	anti-cancer gene delivery	HepG2	[69]
Galactosylated chitosan	5-fluorouracil	anti-cancer	-	[70,71,72]
Galactosylated chitosan	adenosine triphosphate	anti-cancer	HepG2	[73]
Galactosylated chitosan–polycaprolactone	curcumin	anti-cancer anti-oxidant	HepG2	[74,75]
Galactosylated-carboxymethyl chitosan	magnetic iron oxide	theranostics	-	[76]
Galactosylated chitosan	triptolide	anti-cancer anti-inflammatory	-	[77]
Galactosylated chitosan	gemcitabine	anti-cancer antimetabolite	-	[78]

**Table 3:** Glycyrrhizin/glycyrrhetinic acid chitosan nanoparticles for the treatment of HCC and liver-targeting: the therapeutic/theranostic effect and cell cultures used in the study are reported for each reference.

Carrier	Payload	Effect	Cell Culture	Reference
Glycyrrhizin chitosan	-	liver targeting	hepatocytes	[81]
Glycyrrhizin chitosan	lamivudine	anti-retroviral	-	[82]
Glycyrrhizin chitosan	atorvastatin	reduction in hepatotoxicity	-	[83]
Glycyrrhetinic acid chitosan/PEG	doxorubicin	anti-cancer	H22; QGY-7703	[84]
Glycyrrhizin N-caproyl chitosan	-	liver targeting	H22; SMMC-7721	[85]
Glycyrrhizin O-CM chitosan	paclitaxel	anti-cancer	SMMC-7721	[86]
Glycyrrhetinic acid galactose chitosan	mouse IP-10 gene	liver targeting	-	[87]

**Table 4:** Nanoparticles made of chitosan derivatives for the treatment of HCC and liver-targeting: the therapeutic/theranostic effect and cell cultures used in the study are reported for each reference.

Carrier	Payload	Effect	Cell Culture	Reference
Folate-conjugated chitosan	IP-10 gene	anticancer gene therapy	-	[88]
Folate-conjugated chitosan	IP-10 gene	anticancer gene therapy	H22	[89]
Folated poly(ethylene glycol)-chitosan-graft-polyethylenimine	plasmid DNA	anticancer gene therapy	-	[90]
Biotinylated chitosan	plasmid DNA	anticancer gene therapy	SMMC-7721	[91,92,93]
Biotin or biotin/avidin conjugated chitosan	trans-resveratrol	anticancer antioxidant	-	[94]
N,N,N-trimethyl chitosan	monoclonal antibodies	anticancer	HepG2	[95,96]
N,N,N-trimethyl chitosan	lactosyl-norcantharidin	anticancer	-	[97,98,99]
2-chloroethylamine HCl/N,N-dimethyl-2-chloroethylamine HCl combined chitosan	maidkin-siRNA	anticancer gene therapy	-	[100]
N-succinyl chitosan	siRNA and doxorubicin	anticancer gene therapy	-	[101]
Lactose marital carboxymethyl chitosan	adriamycin	anticancer	HU7; H22	[102,103]
Glycol Chitosan-5β-Cholanic Acid	-	tumor targeting	HT29	[104]
